# Evaluating and comparing critical thinking skills of residents of Tehran University of Medical Sciences

**DOI:** 10.1186/s12909-023-04094-7

**Published:** 2023-02-27

**Authors:** Saeed Reza Mehrpour, Amin Hoseini Shavoun, Azita Kheiltash, Rasoul Masoomi, Roya Nasle Seraji

**Affiliations:** 1 Department of Orthopedics, School of Medicine, Tehran University of Medical Sciences, Tehran, Iran; 2 Department of Medical Education, School of Medicine, Tehran University of Medical Sciences, Tehran, Iran; 3 Department of Community Medicine, Tehran University of Medical Sciences, Tehran, Iran; 4 Center for Educational Research in Medical Sciences, School of Medicine, Iran University of Medical Sciences, Tehran, Iran; 5 Shariati Hospital, School of Medicine, Tehran University of Medical Sciences, Tehran, Iran

**Keywords:** Critical thinking, California Critical Thinking Skills Test-Form B (CCTST-B), residents

## Abstract

**Background:**

Today’s complexities and diversity in the clinical setting have revealed the need to pay attention to strengthening critical thinking (CT) skills. The present study aimed to evaluate and compare CT skills in the residents of the Tehran University of Medical Sciences.

**Methods:**

This is a cross-sectional study. The study's statistical population included 284 residents in orthopedic, internal medicine, and surgery groups studying in the PGY1 to PGY4 years of residency. The data collection tool was the California Critical Thinking Skills Test (CCTST) form B. The collected data were entered into SPSS-16 software and analyzed using descriptive (mean and standard deviation) and inferential (one-way ANOVA) statistics. The significant level in all tests was considered at *P* < 0.05.

**Results:**

189 out of 284 residents completed and returned the questionnaire, and the response rate was 66%. The mean CT skill score of residents (M = 13.81, SD = 3.52) was lower than the optimal level (M = 17.1 SD = 5.0). Comparing the mean CT skill scores of the residents separately for the residency year revealed a significant decrease in CT scores in the 4 years. A significant difference was found between the CT skill scores in the three groups (internal medicine, general surgery, and orthopedic surgery).

**Conclusion:**

The CT skills of the residents of Tehran University of Medical Sciences were generally below the optimal level. The CT score of the residents show an increase in PGY2, but a decrease in PGY3 and PGY4. Due to the emphasis of accreditation institutions, the World Federation for Medical Education, and other international educational institutions on the importance of critical thinking, it is recommended to pay more attention to the factors related to the promotion and development of CT skills in residency programs.

## Introduction

Having the ability to critically thinking is a very valuable tool for medical students who, after graduation, are responsible for serious responsibility in the health system as health team leaders. Considering the importance of critical thinking skills, teaching critical thinking skills has become one of the most important activities of universities in Iran today. During the last two decades, major universities of medical sciences in Iran such as Tehran, Shaheed Beheshti, Iran, Isfahan, Tabriz, and Shiraz have taken basic steps to revise the general medicine program. In these universities, critical thinking skills are taught longitudinally and integrated into the phases of basic sciences, physiopathology, clerkship, and internship [1–4]. However, there is no formal and standardized training for teaching critical thinking in residency programs.

Medical care is prone to diagnostic errors. Therefore, it requires the highest possible skills in evidence-based diagnosis and treatment. Misdiagnosis of disease by residents is a type of cognitive error. This error results from incomplete data collection, misinterpretation of data, insufficient reasoning, or incomplete knowledge [5]. Approximately one-third of patient problems are not appropriately managed due to misdiagnosis [6]. Previous researches consider critical thinking (CT) abilities as part of the solution that can reduce the risk of clinical errors [7, 8]. CT can help medical students and healthcare professionals by avoiding medical/clinical errors, Identifying better alternate options for diagnosis and treatment, Better clinical decision-making, and Avoiding litigations [8].

CT is an essential skill for medical residents to be able to make correct decisions, judgments, and reasoning about patient problems in critical and complex situations [9, 10]. CT is a cognitive process to identify and analyze problems and seek and evaluate relevant information to reach an appropriate conclusion [8]. CT includes various skills, the most important of which include: analysis, evaluation, inference, deductive reasoning, and inductive reasoning [5, 11]. Residents need to be able to think critically when dealing with challenging medical issues, such as diagnosis, deciding on treatment plans, and avoiding mistakes [12]. In this regard, the “World Federation for Medical Education” has introduced CT as one of the basic standards of medical education [13]. The Institute for International Medical Education (IIME) has also introduced CT as one of the seven main areas in medical education [14].

Despite the importance of critical thinking, the results of studies suggest the student’s ability to use CT skills is at a moderate level [15]. The American Higher Education Association reported that less than 6% of graduate students achieve CT skills [16]. In a systematic review, the level of CT skills and their tendency toward it among Iranian medical students was reported to be low [17]. Another study has found that the level of CT of residents is lower than the optimal level [18]. Various factors can play a role in justifying this problem. However, inadequacy and weakness in the planning system and educational policies, inefficient curricula, leadership and management methods governing the current educational institutions, lack of proper and sufficient use of active learning methods by medical faculties, lack of student motivation, pressure work, and use of traditional evaluation method, are considered the main factors that interfere improvement of CT over the years [19–21]. A recent study mentioned several challenges and Barriers to students thinking critically: perceptions, poor metacognitive skills, a fixed mindset, heuristics, biases, and because thinking is effortful [22].

Several studies Have examined the factors affecting the improvement of CT skills, such as the level of clinical experiences and observations, educational level, clinical setting, and active learning strategies and learning styles. This studies indicated that students with higher educational levels have more vital positive CT skills and can better meet the needs of health care [23, 24]. Researchers have indicated that the level of clinical experiences and observations is one of the effective factors in the development and improvement of students’ CT skills [25, 26]. The impact of the clinical setting, learning atmosphere, and active learning strategies such as (team-based learning [27], flipped classroom approach [28], problem-based learning [29], and Concept Mapping Education [30]) have been examined in the literature. In addition, the relation correlations between active learning strategies and learning styles with CT is demonstrated in meta-analysis and systematic review studies [31, 32]. Several studies demonstrated the relationship between the clinical environment and CT [33–35].

However, fewer studies have addressed the effect of education level, experience level, and clinical observations on critical thinking [36]. Also, limited studies have evaluated CT skills in residency programs [5, 18, 37, 38]. There is some work being done to promote CT in Medical schools in Iran, where the situational judgment test incorporates the Programme assesses constructs closely related to critical thinking [39]. In a recent decade, TUMS commenced developing and implementing a newly revised curriculum for delivering undergraduate medical education. A main feature of the revised curriculum is that it focuses more attention on the integration of critical thinking programs for training and assessing medical students [2]. However, not much work has been done in this field at the assistantship level. Furthermore, no study has examined CT skills in Tehran University of Medical Sciences residents. Therefore, this study aimed to examine the CT skills of Tehran University of Medical Sciences residents (internal medicine, surgery, and orthopedics). In this study, we compared the results of the California Critical Thinking Skills Test (CCTST) and sub-skills with the post-graduate year, and academic discipline.

## Methods

### Study design

A cross-sectional study was conducted to evaluate and compare residents’ CT skills scores at the Tehran University of Medical Sciences (TUMS). A self-reported questionnaire was used to collect data. The study included the residents in training in the three largest teaching hospitals in TUMS (Imam Khomeini Hospital, Shariati Hospital, and Sina hospital).

### Sampling

The group of the study is 284 residents in training (PGY1, PGY2, PGY3, and PGY4) and were three disciplines of internal medicine, surgery, and orthopedics. After obtaining informed consent, the questionnaire was distributed to all residents starting the residency program (*N* = 284). Except for the assistants who did not meet the inclusion criteria. Inclusion criteria included that the resident is: (1) Spent at least 6 months of residency, (2) willing to participate and complete the questionnaire. (see Table 1).

**Table 1:** Distribution of teaching hospitals residents and their grades

Residency year		PGY1	PGY2	PGY3	PGY4	Sum	Total
Field/Hospital							
Orthopedics	Sina	4	4	4	3	15	59
	Shariati	4	4	4	3	15	
	Imam	7	8	7	7	29	
Total		15	16	15	13	59	
Internal medicine	Sina	6	5	–	–	11	159
	Shariati	15	12	21	20	68	
	Imam	22	12	24	22	80	
Total		43	29	45	42	159	
Surgery	Sina	6	7	6	6	25	66
	Shariati	5	5	4	6	20	
	Imam	5	4	7	5	21	
Total		16	16	17	17	66	
		74	61	77	72	284	

#### The rationale for the different groups

In the present study, it was assumed that internal medicine, surgery, and orthopedics groups have different forms of critical thinking in their specialties. From one side, CT is a general skill set that spans disciplines. Furthermore, it is a specific skill set that varies across disciplines [40]. From the viewpoint of Fero and Ozturk, the level of clinical experience and educational level is one of the effective factors in the development and improvement of students’ CT skills [25, 26]. Previous studies indicated that students with higher educational levels have more vital positive CT skills and can better meet the needs of healthcare [23, 24]. Therefore, one of the aims of the current study was to investigate the status of critical thinking skills based on the different year groups in residents.

Also, in justifying why these specialties were chosen, we can point to the large number of residents accepted in the fields (internal medicine and surgery) and the availability of orthopedic residents due to the compatibility with the specialized field of the project manager.

In Iran, applicants for residency are general Medical Doctorate (MD) who have participated in the central entrance exam and choose their field according to their score and interest. Based on the healthcare priorities, the annual capacity of the residency program is nearly 4300 residents in 35 medical universities in Iran. The length of the training programs is similar to other countries and ranges from 3 to 5 years depending on the types of specialties which is more or less similar to most residency programs worldwide though there are some differences [41]. The chosen specialties in this study were all 4 years.

### Study tool

In this study we use the California Critical Thinking Skills Test (CCTST) form B. The used questionnaire consists of two sections: the demographic characteristics of residents and the California Critical Thinking Skills Test (CCTST). In the pilot study, the validity and reliability of the test were re-examined. Accordingly, to examine the validity, the desired tool was submitted to 12 medical education experts, clinical professors, and interested residents. After receiving their opinions, experts made the necessary corrections and finally approved to examine the reliability of the test, the questionnaire was piloted on 22 residents from different hospitals and specializations. The Cronbach’s Alpha Reliability Coefficient for this questionnaire was 0.81. Facione and Facione specified that the internal consistency reliability (Kuder Richardson-20) of the CCTST was r = 0.70 [42].. The validity and reliability of the Persian translation of this questionnaire have been confirmed in previous studies in Iran [11, 43]. Based on the international expert consensus definition of critical thinking skills as defined by the APA Delphi Report, the California Critical Thinking Skills Test (CCTST) was designed by Facione to measure critical thinking skills in college students [44]. The CCTST is a 45-minute standardized test that includes 34 multiple-choice items assessing five critical thinking domains: analysis (9 items), evaluation (14 items), inference (11 items), deductive reasoning (16 items), and inductive reasoning (14 items) [42]. Each item is presented with four or five response options and one correct answer [44]. The CCTST is dichotomous (correct answer = 1 and incorrect answer = 0); therefore, scores can range from 0 to 34. Higher CCTST scores reveal stronger critical thinking skills.

The Insight Assessment Measuring Thinking Worldwide has determined the optimal CCTST mean for university levels (M = 17.1) [45]. According to this guide the optimal mean for university level CCTST total score in the range 0 to 7 do not manifest evidence of critical thinking. Scores in the range of 8–12 are considered Weak; scores in the 13–18 range are Moderate scores, and scores from 19 to 23 are considered Strong. Scores of 24 or higher are considered Superior. Analysis scores in the range of 0 to 2 do (not manifest) evidence of critical thinking. Scores in the 3–4 range are Moderate scores, scores from 5 or higher are considered Strong. Evaluation scores in the range of 0 to 3 do (not manifest) evidence of critical thinking. Scores in the 4–7 range are Moderate scores, scores from 8 or higher are considered Strong. Inference scores in the range of 0 to 5 do (not manifest) evidence of critical thinking. Scores in the 6–11 range are Moderate scores, scores from 12 or higher are considered Strong. Deduction scores in the range of 0 to 5 do (not manifest) evidence of critical thinking. Scores in the 6–11 range are Moderate scores, scores from 12 or higher are considered Strong. Induction scores in the range of 0 to 5 do (not manifest) evidence of critical thinking. Scores in the 6–11 range are Moderate scores, scores from 12 or higher are considered Strong. The optimal score for total CT skills and subdomains is mentioned in Table 2 [45].

**Table 2:** The optimal score of total CT skills and subdomains

Skill/Attribute Name	OVERALL	Analysis	Evaluation	Inference	Deduction	Induction
**Mean**	17.1	3.7	4.6	8.8	7.5	9.6
**Std. Deviation**	5.0	1.4	2.1	2.6	2.9	2.7

This means that a score lower than the cut-off point indicates weakness in CT skills, and a score higher than that indicates strength and high CT skills [46]. This study considered the above criteria to measure the norm or abnormality of the residents’ CT scores.

#### The rationale for Using the CCTST

Critical thinking is widely recognized as an essential competency in medical education. Still, there is little agreement on how it should be assessed in residency programs [47]. As varied as the definitions and teaching methods are, this is also true about the tools used to measure critical thinking outcomes. There is no gold standard across these studies [48]. For objective standardized measures, used the California Critical Thinking Disposition Inventory (CTDI) [49–51]. They additionally use the California Critical Thinking Test (CCTST). which measures critical thinking skills applied to scenarios (e.g., inference) [49–51]. Razeghi et al. use the Self-Reflection Insight Scale (SRIS), for self-reflection, and insight [52]. Hong and Yu use the Watson & Glaser CT Appraisal (WGCTA) [51]. Shin et al. used Yoon’s CT Disposition tool [53]. CT standardized tests are one of the most popular tools used to assess CT and are widely used in health professions students. CCTST is a famous instrument in this field that measures cognitive and meta-cognitive skills associated with CT [54]. which appears to have the potential for use in residency education [10]. The CCTST (Form B), predicts strength in critical thinking skills in authentic problem situations and success on professional licensure examinations. It also provides an objective measure of CT skills. This test is suitable for college-level and post-baccalaureate student populations [47]. As regards the aim of this study was to evaluate the residents’ CT skills, and the domains (analysis, evaluation, inference, induction, and deduction). In this study, we intended to assess more than one aspect of critical thinking. Therefore, we found that the CCTST test is more comprehensive than the others.

### Data collection

The names of all residents with their mobile numbers have been obtained in each hospital separately. The questionnaire was given to the residents in person. The residents were informed of the purpose of the study and were invited to provide consent to participate at the outset of the survey, therefore, participation was voluntary. A week later, a reminder was sent to the residents who had not responded for the first time.

### Data Analysis

All collected data were analyzed using Statistical Package for Social Sciences (SPSS) Statistics version 16. The descriptive statistics were calculated, including mean, standard deviations, frequencies, and percentages. To examine the normality of quantitative data distribution in different groups, the Kolmogorov-Smirnov normality test was used. For more than two groups, if the data met the normal distribution and the variance was homogeneous, one-way analysis of variance (ANOVA) was used for inter-group comparison, while the LSD method was used for after-the-fact comparison; otherwise, the Kruskal-Wallis test was used. We used a one-sample t-test to compare the mean of a single population to a standard value and the independent sample t-test for compares the mean of CT skills and subdomains by gender. Pearson’s test was conducted to study the correlation between some variables. A *p*-value of < 0.05 was considered statistically significant.

## Results

Out of 284 questionnaires distributed among the residents, 189 of them answered, and the response rate was 66.54%. Among 189 residents, 113 (59.7%) were male and 76 (40.2%) were female. The number of internal medicine, surgery, and orthopedic residents was 88 people (46.5%), 54 people (28.5), and 47 people (24.8%), respectively. They were practicing as a resident in the teaching hospitals of Imam Khomeini (46.03%), Shariati (35.9%), and Sina (17.9%). Among the studied residents, 45 people (23.8%) were studying in PGY1, 58 people (30.6%) in PGY2, 48 people (25.4%) in PGY3, and 38 people (20.1%) in PGY4. In this study, the mean age of residents was 30.29 ± 2.43 years (see Table 3).

**Table 3:** General demographic characteristics of residents (*n* = 189)

Variable	n	%
Gender		
Female	76	40.2
Male	113	59.8
Year in residency training		
PGY1	45	23.8
PGY2	58	30.7
PGY3	48	25.4
PGY4	38	20.1
Marital status		
Married	134	70.9
Single	55	29.1
Specialty		
Internal medicine	88	46.6
Surgery	54	28.6
Orthopedics	47	24.9
Age (years)		
25	2	1.1
26	6	3.2
27	17	9.0
28	18	9.5
29	30	15.9
30	38	20.1
31	23	12.2
32	21	11.1
33	14	7.4
34	8	4.2
35	8	4.2
36	2	1.1
37	2	1.1

A sample t-test was conducted to compare the residents’ critical thinking mean with the expected mean. The results showed that there is a significant difference between the residents’ critical thinking mean and the expected mean (M = 17.1, SD = 5.0). The residents’ critical thinking mean was lower than the expected mean (M = 13.81, SD = 3.52), t (189) = − 12.811, *p* = .000. (see Table 4).

**Table 4:** One-sample t-test for Critical thinking skills (Test Value = 17.1)

CCTST Total	N	Mean	Std. Dev.	CI for μ	t-test	*p*-value
	189	13.814	3.525	−3.791, −2.779	−12.811	.000

An Independent sample t-test was conducted to compare the residents’ critical thinking mean by gender. The results showed that there is a significant difference between the residents’ critical thinking means and the gender group. t (189) = − 1.644, *p* = .008. (see Table 5).

**Table 5:** Independent sample t-test for Critical thinking and subscales by gender (male *n* = 113, Female *n* = 76)

Scale	Gender	Mean	Std. Deviation	t-test	*P*-value
Analysis	Male	3.5929	1.70418	.112	.214
	Female	3.5658	1.52609		
Evaluation	Male	5.0885	1.97103	−1.790	.044
	Female	5.5789	1.64327		
Inference	Male	4.8053	2.04790	−1.203	.619
	Female	5.1579	1.86228		
Deductive	Male	6.9292	2.22693	−1.230	.876
	Female	7.3289	2.13784		
Inductive	Male	5.1593	2.21427	−1.407	.044
	Female	5.5921	1.84158		
Total CCTST	Male	13.4867	3.82687	−1.644	.008
	Female	14.3026	2.98002		

The Pearson correlation coefficient was used to determine the correlation between residents’ CT scores with their age, gender, educational Level, and marital status. The result showed a weak positive correlation between the age with subscales of evaluation (r = 0.209, *p* = 0.004), and the inductive reasoning, (r = 0.160, *p* = 0.028. (see Table 6).

**Table 6:** Correlation between residents’ characteristics, Critical thinking, and subscales

Variables	Analysis		Evaluation		inference		Deductive reasoning		Inductive reasoning		Total CT skills	
	r	p	r	p	r	p	r	p	r	p	r	p
Age (years)	−.094	.197	**.209**^a^	**.004**	−.010	.890	−.024	.746	**.160**^b^	**.028**	.061	.407
Gender	−.008	.911	.130	.075	.088	.230	.090	.220	.102	.161	.114	.119
Educational Level	−.083	.257	.125	.086	.036	.624	.018	.806	.105	.149	.048	.514
Marital Status	−.036	.624	.084	.253	−.089	.223	−.122	.094	.105	.151	−.023	.758

^a^Correlation is significant at the 0.01 level (2-tailed)

^b^Correlation is significant at the 0.05 level (2-tailed)

**Fig. 1:**
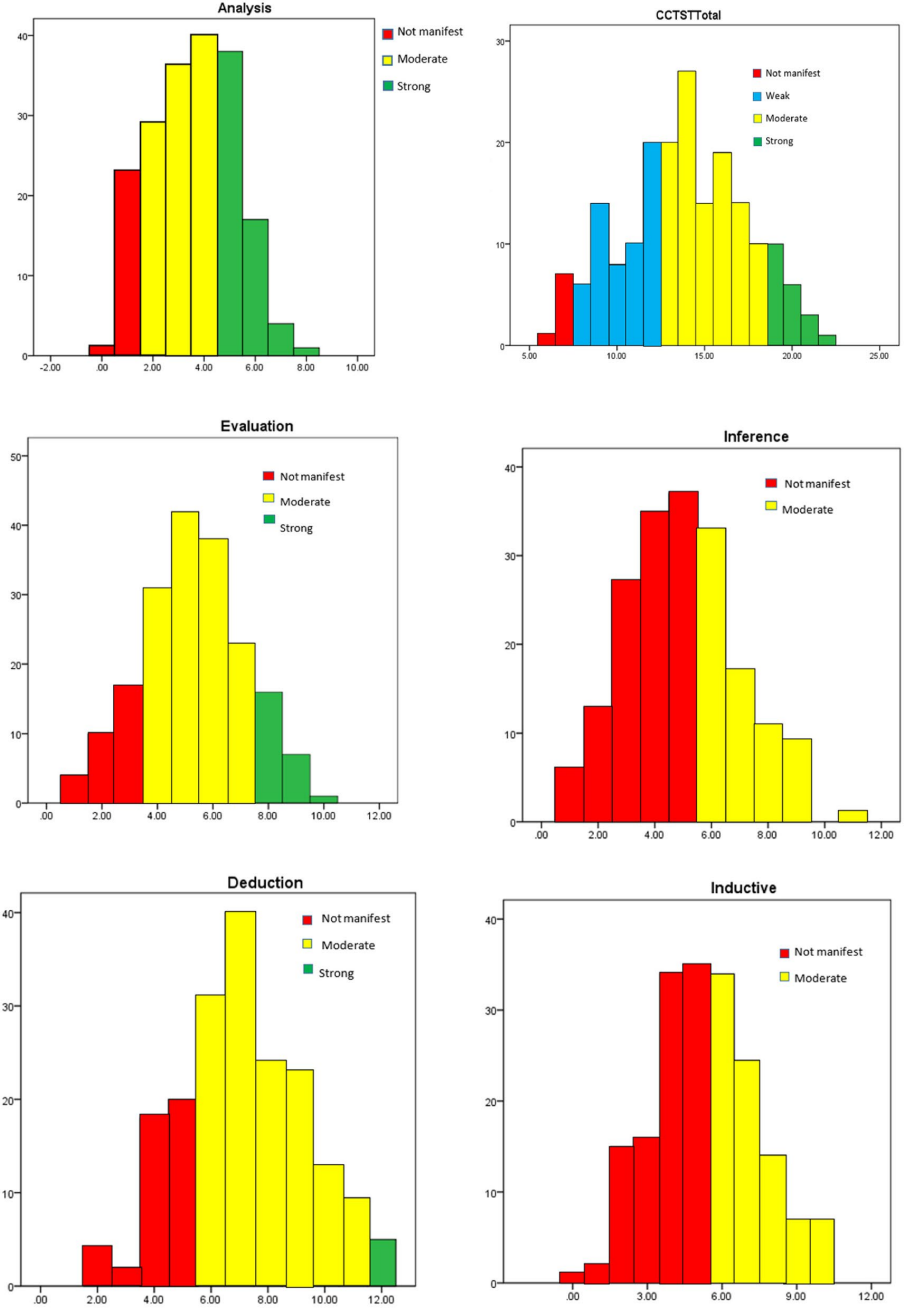
Total critical thinking and subdomains scores

The results showed that residents’ CT Skills mean score (M = 13.81, SD = 3.52) is weak and below the optimal average (M = 17.1 SD = 5.0). Also, the residents’ CT Skills mean score in all subdomains was lower than the cut-off point. ((see Fig. 1 and Table 7).

**Table 7:** Total critical thinking score and subdomains

Skill/Attribute Name		OVERALL	Analysis	Evaluation	Inference	Deduction	Induction
**N**		189	189	189	189	189	189
**Mean**		13.81	3.58	5.28	4.94	7.08	5.33
**Median**		14.00	4.00	5.00	5.00	7.00	5.00
**Std. Deviation**		3.52	1.63	1.85	1.97	2.19	2.07
**SE Mean**		0.25	0.11	0.13	0.14	0.15	0.15
**Minimum**		6.00	.00	1.00	1.00	2.00	.00
**Maximum**		22.00	8.00	10.00	11.00	12.00	10.00
**Percentiles**	**25**	12.00	2.00	4.00	4.00	6.00	4.00
	**50**	14.00	4.00	5.00	5.00	7.00	5.00
	**75**	16.00	5.00	6.50	6.00	9.00	7.00

One-way analysis of variance (ANOVA) was performed among the groups to compare the mean scores of CT skills of orthopedic, surgical, and internal medicine specialist residents. There was a statistically significant difference between the CT skill scores for the three groups (F = 12.3 at the level of *P* = 0.05). due to the equality of the variance of the populations and the significance of the F test, the post hoc comparisons using the LSD test showed that the mean scores of the internal medicine group (SD = 3.45, M = 14.48) had a significant difference with the orthopedic group (SD = 3.71, M = 13.10). The surgery group (SD = 3.33, M = 13.33) did not have a significant difference from the internal medicine and orthopedic groups (see Table 8).

**Table 8:** Comparison of critical thinking and subscales scores by educational groups

Scale	Internal medicine	Surgery	Orthopedic	F	*P*-value
	Mean ± SD	Mean ± SD	Mean ± SD		
Analysis	1.58 ± 3.80	1.55 ± 3.35	1.77 ± 3.42	1.60	0.205
Evaluation	1.81 ± 5.46	1.93 ± 5.20	1.84 ± 5.04	0.868	0.421
inference	2.06 ± 5.21	1.90 ± 4.77	1.85 ± 4.63	1.59	0.206
Deductive reasoning	2.23 ± 7.60	2.09 ± 6.62	2.06 ± 6.65	4.66	0.011
Inductive reasoning	2.03 ± 5.42	1.96 ± 5.31	2.29 ± 5.19	0.187	0.816
Total	3.45 ± 14.48	3.33 ± 13.33	3.71 ± 13.10	3.12	0.046

Also, a one-way analysis of variance (ANOVA) among the groups was performed to examine the mean score of CT skill domains of residents’ groups. There was a statistically significant difference between deductive reasoning factor scores for the CT sub-factors (F = 66.4 at the level of *P* = 0.05). Due to the equality of the variance of the populations and the significance of the F test, post hoc comparisons using the Scheffe test showed that the mean scores of the surgery group (SD = 2.23, M = 7.60) were significantly different from the surgery group (SD = 2.09, 6.62). There was no significant difference between the surgery group residents and orthopedic group residents in terms of mean scores of deductive reasoning (see Table 8).

**Fig. 2:**
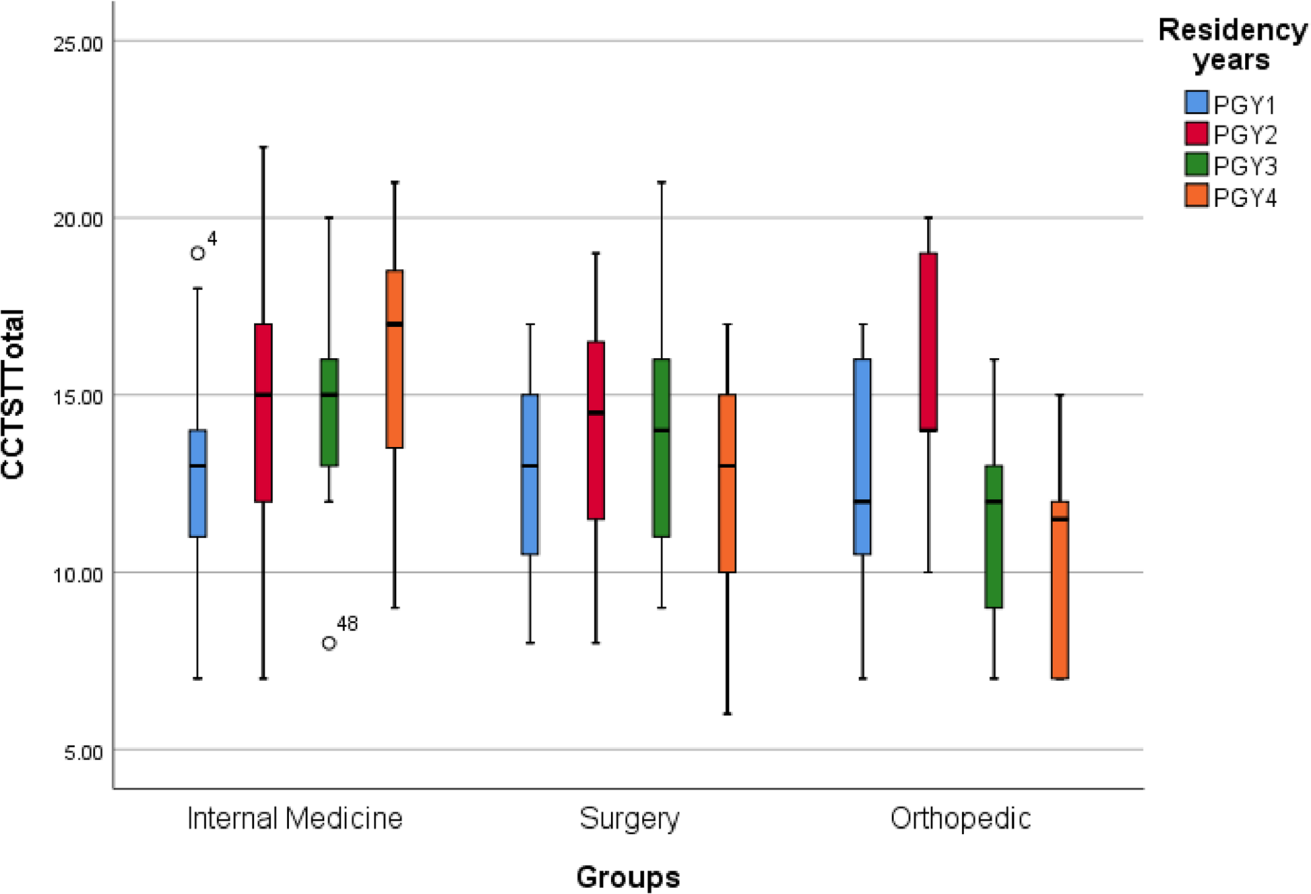
Trend of critical thinking and subscales scores by residency year

One-way analysis of variance (ANOVA) among the groups was performed to compare the mean scores of CT skills of residents of orthopedic, surgery, and internal medicine groups separately for CT factors and year of residency. There was a statistically significant difference between the scores of CT score from PGY1 to PGY4 (F = 3.09 at the level of *P* = 0.05). Due to the equality of the variance of the populations and the significance of the F test, posthoc comparisons using the Scheffe test showed that the mean scores of the residents in the PGY1(SD = 3.19, M = 12.76) had a significant difference from the PGY2(SD = 3.46, M = 14.83). The mean scores of the PGY2 residents (SD = 3.46, M = 14.83) were not significantly different from the PGY3 and PGY4 residents. The detailed information is shown in Table 9; Fig. 2.

**Table 9:** Comparison of critical thinking and subscales scores by residency year

Scale	PGY1	PGY2	PGY3	PGY4	F	*P*-value
	Mean ± SD	Mean ± SD	Mean ± SD	Mean ± SD		
Analysis	1.38 ± 3.24	1.71 ± 4.17	1.54 ± 3.66	1.60 ± 2.97	5.32	0.002
Evaluation	2.02 ± 4.88	2.02 ± 5.27	1.61 ± 5.45	1.65 ± 5.55	1.08	0.365
inference	1.73 ± 4.62	2.10 ± 5.37	1.72 ± 4.52	2.21 ± 5.21	2.34	0.074
Deduction	1.72 ± 6.55	2.13 ± 7.67	2.05 ± 7.02	2.76 ± 6.92	2.39	0.074
Induction	2.19 ± 4.86	2.11 ± 5.51	2.03 ± 5.29	1.92 ± 5.65	1.22	0.301
Total	3.19 ± 12.76	3.46 ± 14.83	3.25 ± 13.64	3.99 ± 13.73	3.09	0.028

Also, a one-way analysis of variance (ANOVA) among the groups was performed to compare the mean scores of CT skills of subgroups. There was a statistically significant difference between the scores of CT skills for the analysis sub-factor according to the residency year (F = 32.5 at the level of *P* = 0.05). Due to the equality of variance of populations and the significance of the F-test, post hoc comparisons using the Scheffe test showed that the mean score of the PGY1 residents (SD = 1.38, M = 3.24) was significantly different from the mean score of PGY2 residents (SD = 1.71, M = 4.17). The mean score of PGY2 residents had a significant difference from the mean score of PGY4 residents (SD = 1.60, M = 2.93), but there was no difference from the mean score of PGY3 residents The detailed information is shown in Table 9; Fig. 2.

## Discussion

The present study aimed to investigate the status of residents’ CT skills during training at the Tehran university of medical sciences. The results showed that residents’ CT skills mean score (M = 13.81, SD = 3.52) is weak and below the optimal average (M = 17.1 SD = 5.0). It seems, the residents’ CT skills mean score at Tehran medical school is not favorable. This finding is consistent with previous study conducted in Iran that reported the CT level of residents as weak [18].

Also, the residents’ CT skills mean score in all sub-skills was lower than the cut-off point, (mentioned in methodology [44]). a score lower than the cut-off point indicates weakness in CT subdomains, and a score higher than that indicates strength and high CT skills [46]. By comparing the obtained results with an optimal mean score, it seems the obtained mean score for all subdomains is lower than the expected score. In a study conducted by Leach (2011) on 1502 students of different faculties in the United States, the mean scores of analysis (M = 4.56, SD = 1.34), evaluation (M = 4.80, SD = 2.70), inference (M = 7.92, SD = 2.51), deductive reasoning (M = 7.33, SD = 2.87) and inductive reasoning (M = 9.94, SD = 2.66) were obtained at higher levels compared to the present study [55]. Ross et al. showed that residents performed better than practicing physicians in nearly all aspects of CT. Age was the strongest predictor of CT skills in practicing physicians [37]. A study conducted in Brazil shows that the student’s ability to use CT skills is at a moderate level [15].

It can be concluded that the residents’ mean score of subdomains CT, compared to the results obtained from the studies, as well as the expectation from higher educational levels such as the residency level, the obtained means, seem to be lower than expected. Studies point to several intertwined factors giving rise to the poor level of residents’ CT skills, including traditional educational system, work pressure, lack of interactive teaching methods, emphasis on memorization, lack of student motivation, and lack of appropriate techniques for cultivating CT skills [10, 33, 56]. Given that CT is an essential skill for medical residents to be able to make correct decisions, judgments, and reasoning about patient problems in critical and complex situations [9, 10], it is imperative to pay attention to cultivating and promoting these skills in the residency program. Several studies suggest that teaching approaches such as PBL [57], team-based learning [58], simulation [59], flipped classroom approach [28], Concept Mapping Education [30], and metacognition [60] can increase overall CT skills.

Other findings of the present study demonstrated a significant difference between residents’ CT and gender groups. The average score of CT skill in women was slightly higher than that of men. In the sub-domains, there was no difference between the mean scores of CT skills in men and women. The results of this study are similar to the findings of Hosseini et al. In their study, it was found that there is no difference between CT sub-skills between men and women [11]. While in the study of Sharifinia et al., the level of CT and the subscales of evaluation and deductive reasoning were significantly higher in male students compared to female students [10]. The reason for this can be the difference in learning styles between men and women. Also, the current study showed a weak positive correlation between age with evaluation and inductive reasoning. Between the gender, educational level, and marital status with CT skill no correlation was found.

Comparing the mean scores of CT skills of residents by the residency years showed that the PGY2 residents have the highest and the PGY1 residents have the lowest CT skill score. The most striking finding of the study is that there is a significant difference between the mean scores of CT of residents for the residency year. Post hoc comparisons using the Scheffe test revealed that the mean scores of PGY1 residents were significantly different from the CT mean scores of PGY2 residents. The CT mean scores of PGY2 residents were not significantly different from the mean scores of PGY3 and PGY4 residents. Post hoc comparisons using the Scheffe test showed that the mean scores of the PGY1 residents had a significant difference from the mean scores of the PGY2 residents. The mean scores of PGY2 residents had a significant difference from the mean scores of PGY4 residents, but their mean scores showed no significant difference from the mean scores of PGY3 residents. Also, comparing the mean scores of CT subdomains of residents showed a significant difference between the CT skill scores for the analysis subdomain based on the residency year. In general, the mean scores of sub-skills suggest that the obtained scores are less than 50% of the total scores of the relevant sub-skills.

Although students’ CT skills is expected to increase with their education years, the findings of the present study, in line with many studies, have shown no CT skills increase in medical students during their education years [10, 11, 61]. A study at the University of São Paulo, Brazil (2021) also showed that the students’ CT level was low and did not change at different stages of their education [62]. Yasayi et al. investigated the students’ CT levels from the first year to the end of their studies at the university, and they reported that there was no significant difference between the CT scores from the first year to the last year at the university and the CT level of medical students did not increase during the period in which they were at university [63]. In addition, in a study by Hosseini, it was reported that the CT scores of medical students decreased during their university years [11].

The results appear to suggest that critical thinking does not progress beyond PGY1 as mentioned before various factors are important in this issue. The use of traditional educational strategies in the current curricula and educational system can be a possible reason for residents’ decline in CT skills scores from PGY1 to PGY4. Another factor may be related to the insufficient development of some critical thinking characteristics, such as flexibility and truth-seeking in residents. Finally, it seems that clinical practice guidelines and residents’ annual grading based on assessment have a major impact on their not being interested in CT skills. Several studies have demonstrated the role of the inadequate and weak educational system, inefficient curricula, leadership and management methods, lack of proper active learning methods by medical professors, lack of student motivation, work pressure, and use of traditional assessment methods in this regard [19–21]. Additionally, poor metacognitive skills and a fixed mindset which is developed during previous years of study play a role in declining CT scores. One way to ensure good CT skills in trainees is to select trainees that already demonstrate those skills. To do this, programs could include CT skills assessment during medical school or residency selection. In a recent decade, TUMS commenced developing and implementing a newly revised curriculum for delivering undergraduate medical education. A main feature of the revised curriculum is that it focuses more attention on the integration of critical thinking programs for training and assessing medical students [2].

Comparing the mean scores of CT skills of residents of orthopedic, surgery and internal medicine groups showed that there was a statistically significant difference between the scores of CT skills for the three groups. Post hoc comparisons using the LSD test showed that the mean scores of the internal medicine group were significantly different from the orthopedic group. The surgery medicine group was not significantly different from the internal and orthopedic groups. A review of the literature by the researcher showed that no similar study has been conducted to compare the mean scores of educational groups, and the results of this part of the study are considered innovative. A glance at the educational status of residents in educational centers indicates that a large amount of residents’ time and energy is spent responding to the large volume of visiting patients and the excessive presence of the patient and the problem of their overcrowding severely disrupts the process of education and direct communication between teacher and student in educational and medical centers. Education has become just one of the several duties of professors and residents, along with specialized, consulting, research, and sometimes management services. This problem is more severe in surgical fields, and in addition to spending time in outpatient clinics and inpatient departments, the presence of residents in the operating room during daytime and night shifts is also added to the above factors. An increase in working hours and the problems caused by it is one of the factors that reduce the mental abilities of surgery and orthopedic residents in response to CT test questions.

## Conclusion

The CT skills of the residents of Tehran University of Medical Sciences were generally below the optimal level. The CT score of the residents show an increase in PGY2, but a decrease in PGY3 and PGY4. Due to the emphasis of accreditation institutions, the World Federation for Medical Education, and other international educational institutions on the importance of critical thinking, In the future, it is recommended to pay more attention to the factors related to the promotion and development of CT skills in residents.

### Research Limitations

One of the limitations of this study was the time limitation, and the study was necessarily conducted using a cross-sectional method. Thus, the results might have been influenced by the individual differences of the participants in different years. Therefore, it is recommended to conduct a study with a longitudinal design and investigate the CT skills of the residents from the start of the residency program to graduation. California CT Skills Test (CCTST), which is a general tool, was used in the present study to evaluate the CT ability of residents. One of the limitations was the large number of questions on the questionnaire and the fatigue of the respondents during the administration of the test. Also, due to the standard nature of the questionnaire and the measurement of five sub-skills, it was not possible to eliminate some questions. Thus, to reduce this weakness, it is recommended to use side measures such as giving spiritual gifts and providing favorable conditions for administrating the test. Also, for a more accurate evaluation of the implicit effect of educational and professional experiences on the CT ability of residents, it is recommended to conduct research using specialized tools to evaluate CT skills in specialized fields. Another main limitation of the present study was the impossibility of administrating the test at the same time and place with the presence of all participants due to the work situation and rotating shifts of the residents. To solve this problem, the test was administrated after prior coordination with the residents, and with repeated visits in groups of several people and even a single person. Hence, we tried to provide similar conditions for all residents to participate in the test.

Another limitation of the study was the impossibility to collect more demographic information and provide comprehensive individual information due to the profession of the subjects and their busyness and sensitivity. To prevent non-cooperation and excessive dropout of samples, the demographic information form was revised in several stages and several personal and sensitive questions such as the promotion scores and rank of the residency test, student number, and the name of the medical training centers where the resident studied, which might lead to the identification of the residents and the possibility of their non-cooperation, were eliminated from the questionnaire. Some individual factors such as personality, family, and social characteristics of people, as well as cases such as anxiety, and the level of concentration of the participants during the test influenced the results of the test. To control such psychological conditions, the researcher recommended the subjects not complete the questionnaires in a hurry to collect real information. Based on the literature review by the researcher no study has been conducted in the medical residency period to compare the results of the present study with other studies in this regard. Thus, the results of this study were inevitably compared in the discussion and conclusion section with those of studies conducted at the Ph.D. level of general medicine.

## Data Availability

The datasets used and/or analyzed during the current study are available from the corresponding author upon reasonable request.
